# Full-Mouth Screw-Retained Implant-Supported Rehabilitation with Multiunit Abutments Using Virtual Guided Surgery and Digital Prosthetics Protocol

**DOI:** 10.1155/2020/3585169

**Published:** 2020-09-09

**Authors:** Igor Ashurko, Artem Trofimov, Svetlana Tarasenko, Sabina Mekhtieva

**Affiliations:** ^1^Department of Surgery Dentistry Institute of Dentistry, Federal State Autonomous Educational Institution of Higher Training I.M. Sechenov First Moscow State Medical University of the Ministry of Health of the Russian Federation (Sechenov University), 8-2 Trubetskaya St., 119991 Moscow, Russia; ^2^Dental Clinic(Rio-Stom), 9 Veliyaminovskaya St., 105318 Moscow, Russia

## Abstract

Total rehabilitation is one of the most sophisticated kinds of dental implant-supported prosthetics. The usage of multiunit abutment system allows the clinician an accurate and passive fitting of screw-retained full-arch construction. In addition, it retains a condition of soft and bone tissues around prosthetic construction. The aim of this case is to demonstrate a modern approach in planning and realisation of full-mouth screw-retained dental implant prosthetics. A 59-year-old patient had an extraction of all failed teeth on upper and lower jaws with immediate 16 implant placement (8 implants on maxilla and 8 implants on mandible) using surgical template. Multiunit abutments were installed intraoperatively. Temporary constructions were fixed immediately after surgery. After 3 months of dental impressions, plaster models and their scan were prepared to make final screw-retained zirconium dioxide constructions. Reevaluation of functional and aesthetic result of the treatment was made after 12 months.

## 1. Background

Dental implantation is a well-documented treatment of partial or full adentia [1, 2]. Success indications of total rehabilitation with dental implants are high and directly depend on preoperative planning [3, 4]. Modern CAD/CAM technologies give maximally accurate choice of size and position of dental implants and furthermore allow precise placement according to preoperative planning. However, the choice of cement or screw fixation of prosthetic construction is still a subject of discussion [5–8]. One of the significant problems of full-arch implant-supported prosthesis is to reach the passive fitting. Perfect passive fit is achieved when the opposing surfaces of the implants and the framework intaglio are in maximal spatial congruency, without strains in the components after tightening of all screws, provided the implant and framework surfaces are fabricated perfectly plain [9]. Screw retaining from bone level platform may not secure the passive fitting of construction. Moreover, at this type of fixation, complications may vary from a fracture of various components in implant suprastructure system to an implant fracture or failure of its osseointegration [6, 10]. On the other side, cement retaining can provide passive fitting due to cement space between the abutment and prosthesis, but this type of fixation may provide other complications like the absence of maintenance service possibility and risk of peri-implantitis development because of excess cement in the peri-implant area, more specifically in soft tissues [11–13]. In this regard, the use of multiunit abutment system is an option for making screw-retained implant-supported prosthetic constructions. The multiunit abutments provide absolutely passive fitting of prosthesis even with significant divergence of placed implant axes. Furthermore, intraoperative multiunit abutment placement protects peri-implant soft tissues from damaging by multiple screwing/unscrewing implant suprastructures, because all manipulations will happen above the bone level and implant platform. This clinical case shows predictable treatment protocol of full-arch implant-supported rehabilitation using guided surgery, intraoperative multiunit abutment choosing, immediate loading, and final prosthetics.

## 2. Case Presentation

**Figure 1:**
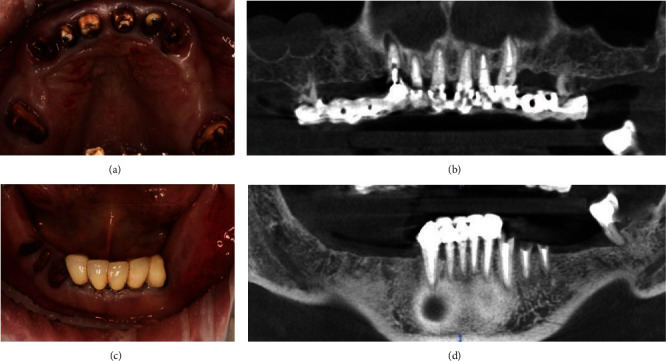
Maxillary (a, b) and mandibular (c, d) teeth condition: multiple carious lesions, absence of ferrule-effect, and periodontal and other changes, which demands for extraction of all decayed teeth.

A 59-year-old male patient came to department of surgery dentistry at the Institute of Dentistry (Sechenov University) complaining about partial teeth absence, difficulty in chewing, and dissatisfaction in teeth aesthetics. According to the patient, he loses his teeth gradually over 20 years due to caries and its complications (Figure 1). Prosthetic constructions were made about 10 years ago.

## 3. Planning

**Figure 2:**
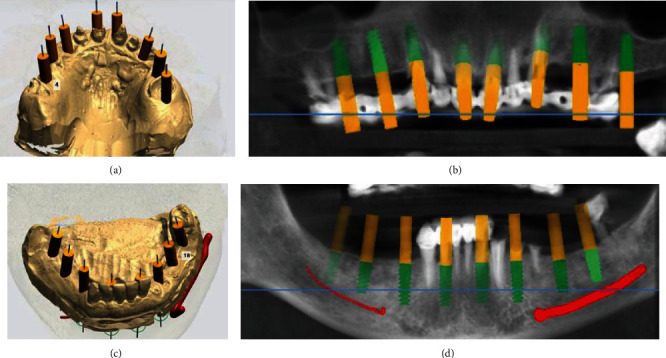
Dental implants positions on maxilla (a, b) and mandible (c, d).

Supporting teeth failure was revealed after cone-beam computed tomography and removal of old prosthetic constructions. The treatment plan was composed under functional and aesthetic analysis, which included the extraction of all teeth with immediate 16 dental implant placement (8 implants for each jaw) and intraoperative multiunit abutments installed with immediate loading. The making of zirconium dioxide fixed screw-retained prosthesis by multiunit abutment level was planned after a period of osseointegration. Received CBCT images and scanned plaster models were exported into programme application Implant Studio (3Shape, Denmark). Placement and sizes of dental implants were chosen after evaluation of bone situation and position of virtual prosthetic construction (Figure 2).

**Figure 3:**
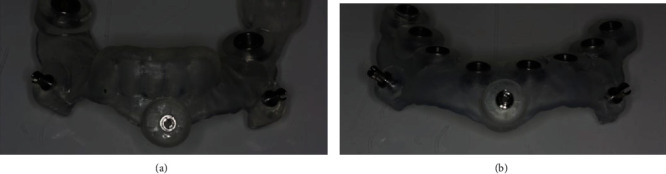
Positioning surgical template (a) and full-guided surgical template (b).

Two surgical templates were made for each jaw. The first template was positioning the teeth-supported surgical template containing sleeves for drilling intraosseous holes for the placement of fixation pins. The localisation of these holes fully matches similarly in the second, full-guided surgical template attached on the edentulous jaw by fixation pins in the same intraosseous holes after extraction of all teeth. Thus, maximum accuracy of the full-guided template was achieved on working on edentulous jaws (Figure 3). The patient reviewed the treatment plan and signed the informed procedure agreement.

## 4. Surgical Procedure

**Figure 4:**
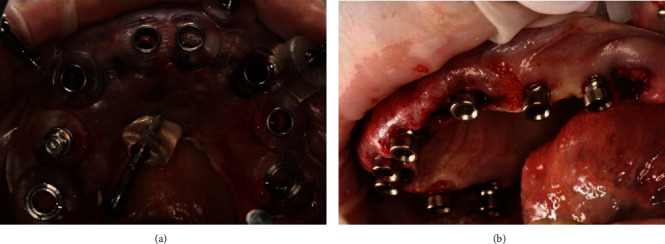
Full-guided surgical template attaching to the upper jaw (a). The condition of the jaw after placement of 8 dental implants by using flapless method with installed implant drivers (b).

The patient received a prophylactic dose of antibiotic (875 mg/125 mg augmentin+clavulanic, GSK GlaxoSmithKline, London, UK) 1 hour before surgery. All procedures were performed with local anaesthetics. Intravenous sedation was patient's request. After local anaesthesia by Ubistesin (articaine 4% with 1 : 200000 adrenaline solution for local anaesthesia, 3 M ESPE, St. Paul, MN, USA), positioning the surgical template was attached on each jaw, and intraosseous holes for fixing pins were drilled to a specified depth, and then, the templates were removed. Afterwards, all of patient's teeth were extracted by using Periotome (Hu-Friedy, USA/Germany) and gentle elevation with forceps to prevent a fracture of the facial alveolar bone. All extraction sockets were thoroughly treated with aggressive curettage. Before immediate implant placement, a decontamination protocol was performed by packing extraction sockets soaked in 0.2% chlorhexidine gluconate gauze and keeping there for 5 min in situ. Then, on each edentulous jaw, the full-guided surgical template was attached tightly by fixing pins to previously prepared intraosseous holes. Placement of 16 SGS dental implants (SGS Dental Implant System Holding, Switzerland) (8 implants on each jaw) was performed under standard protocol. Eight SGS implants were used on the maxilla with aggressive thread (P7), and they were placed transmucosally (flapless). The criteria for the flapless surgery were an adequate amount on the bone for implant placement, the presence of 2 mm thickness each bone on buccal and palatal sides around implant as planned in a favourable prosthetic position (which defined by using CBCT analysis), and the presence of sufficient keratinised attached gingiva (it must be at least 3 mm around the each implant) (Figure 4).

**Figure 5:**
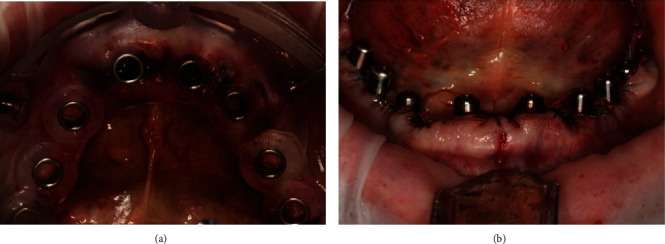
Full-guided surgical template attaching to the mandible (a). The condition of the jaw after placement of 8 dental implants by using standard protocol (b).

SGS implants P1 were used on the mandible with parallel surfaces and placed by forming mucoperiosteal flap and standard protocol drilling, excluding places where extraction sockets were located, as immediate implantation was made there using the flapless method (Figure 5).

**Figure 6:**
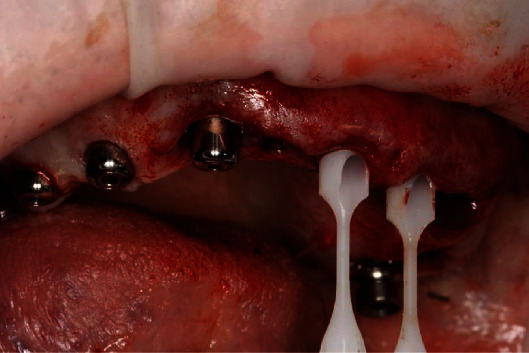
Intraoperative selection of 30 degrees multiunit abutments at the front side of maxilla and installation healing caps at the distal side.

All the implants were placed with minimal torque above 50 Ncm. Afterwards, the selection of multiunit abutments (SGS Dental Implant System Holding, Switzerland) was made intraoperatively. For the four implants in the front of maxilla, two-component 30-degree multiunit abutments were chosen, and left over on the distal side are four direct one-component multiunit abutments. All of these multiunit abutments have the height of the transgingival part equal to 2 mm. For the mandibular implants, direct one-component multiunit abutments were chosen with the height of the transgingival part equal to 1 mm. Thus, all of the multiunit abutments were installed intraoperatively with a torque equal to 25 Ncm, and their removal was not performed in the future (Figure 6).

Then on multiunit abutments were installed healing abutments and made 6-0 Prolene (Ethicon W8005, Johnson & Johnson) monofilament interrupted sutures to close the flaps. Augmentation of extraction sockets by xenograft was not performed. Full-arch maxillary and mandibular impressions were made from multiunit abutment platform with appropriate transfers for open tray with polyether impression material (Impregum, 3 M ESPE, St. Paul, MN, USA) and sent to the laboratory for making first temporary constructions in centric occlusion relationship. The prepared temporary restorations were attached after 4 h with recommended torque equal to 10 Ncm, and target X-rays of the implant suprastructure systems were performed.The patient was instructed for postoperative oral care like rinsing oral cavity with 0.2% (by volume) chlorhexidine gluconate mouthwash (Corsodyl, GlaxoSmithKline) solution twice a day for a week and cleaning the temporary restorations with an extra fine toothbrush. The patient received an antibiotic (1 g amoxiclav, LEK, d.d., Slovenia) twice a day for 5 days. For pain control, the patient was prescribed a 100 mg of nimesulide (Nise; Dr. Reddy's Laboratories Ltd., India). The patient was also advised to minimise trauma at the surgery site; no special diet was recommended. The sutures were removed 10 days after the surgery.

## 5. Prosthetic Procedure

**Figure 7:**
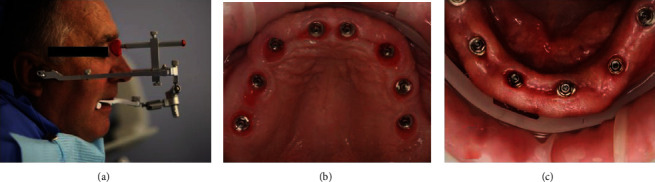
Final prosthetics after 3 month healing (a). Good condition of soft tissues on maxilla (b) and mandible (c) after wearing temporary screw-retained multiunit abutment-supported restorations.

Further prosthetic procedures were initiated after 2 months of soft and bone tissue healing. For centric relation (CR) definition, Kois Deprogrammer was prepared and recommended to wear for 8 h each day for a week. After that, second temporary constructions in CR relationship were prepared. Another month later (3-month postoperative), the impression was taken on a multiunit abutment level with transfers for open tray with polyether impression material (Impregum, 3M ESPE, St. Paul, MN, USA). Temporary prosthetic constructions were installed to prepared plaster models, which were mounted on the articulator SAM 3 (SAM PRÄZISIONSTECHNIK GmbH, Gauting, Germany) (Figure 7).

**Figure 8:**
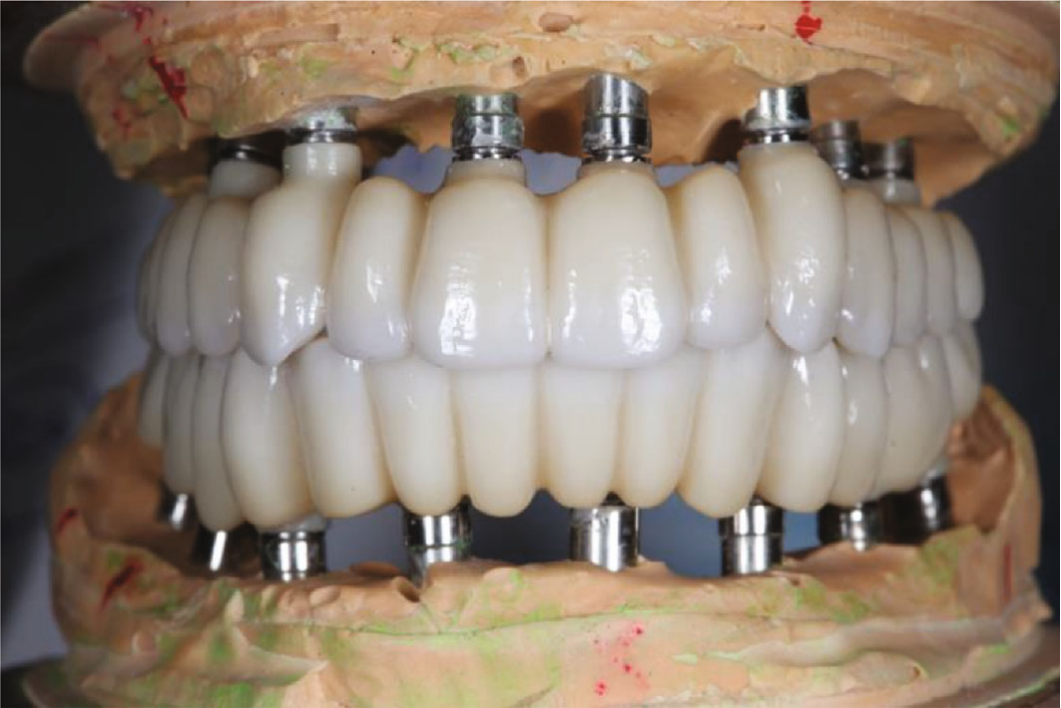
Final look of the zirconia full-arch prostheses. Afterwards, the titanium bases were inserted.

The prostheses were designed on the ExoCAD software (ExoCAD GmbH, Darmstadt, Germany). An epoxy resin prototype was printed and sent to verify the lip support, midline position, incisal edge position and teeth display, occlusal plane orientation, centric relation, phonetics, and aesthetics for the sake of patient's satisfaction. After some adjustments, the restorations in full anatomy were milled in monolithic zirconia block (KATANA Zirconia STML, Kuraray Noritake Dental Inc., Aichi, Japan) (Figure 8) and were sintered, colourised, and then glazed. Each part of restoration was bonded with titanium base in the following ways: both surfaces were treated with 50 microns of aluminium oxide particles at 2 bar pressure (0.25 MPa) for 20 s at a distance of 10 mm (RONDOflex Plus 360, KaVo, Germany). After that, a universal single-component priming agent (Monobond Plus, Ivoclar Vivadent, Liechtenstein) was applied to the zirconia and titanium bases accordingly. Dual-curing luting composite for the aesthetic and permanent cementation of ceramic (Variolink Esthetic DC, Ivoclar Vivadent, Liechtenstein) was used to lute the two components extraorally according to the manufacturer's recommendations.

**Figure 9:**
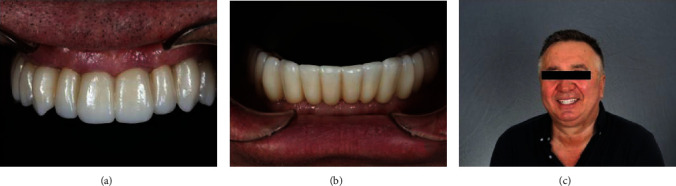
Final look of the prostheses on maxilla (a) and mandible (b). Patient's joyful smile (c).

**Figure 10:**
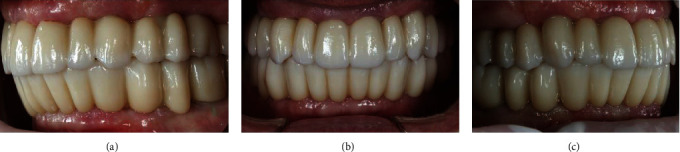
Occluding prostheses: left side (a), front side (b), and right side (c).

The final full-arch prostheses were clinically verified with a one-screw test for passive fitting. The prosthesis screws were tightened following the instructions to 15 Ncm. The screw access holes were filled with temporary material. Functional specificities, including group function, interocclusional contacts, no contacts on protrusive and lateral excursions, were achieved by minor selective modifications, central fossa expansions on premolars and molars and teeth appearance reduction (Figures 9 and 10).

**Figure 11:**
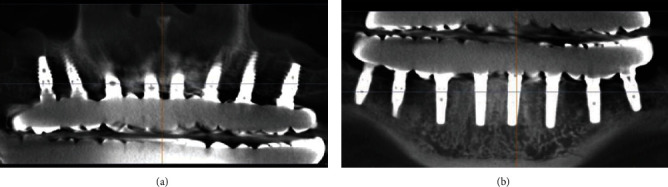
Condition and level stability of the bone tissue around dental implants after 12 month of prosthetics. (a) Is maxilla and (b) is mandible.

Finally, the aesthetics and phonetics were verified. Occlusion check was made by articulation paper 100-micron thickness (BAUCSH, Germany). Three days later, the occlusion recheck was made by articulation paper 48-micron thickness (BAUCSH, Germany). Front teeth in full contact 8-micron disocclusion were achieved. Screw access holes were filled with the flowable resin Filtek Ultimate Flow (3 M ESPE, USA). Postoperative instructions including hygiene care were advised to the patient. For outcome and follow-up, the patient was surveyed after 6 weeks and 12 weeks postoperatively for monitoring the stability of the implants and assessing the health of the peri-implant tissues. Postoperative instructions including hygiene care were advised to the patient. Multidisciplinary regular check-up was emphasised. The patient was followed at 1, 3, 6, and 12 months post loading. CBCT scanning was taken after 12 months, and no any bone resorption around implant platform was found (Figure 11). Soft tissues were completely healthy at the follow-up time.

## 6. Discussion

Zirconium as a material for permanent restorations has many beneficial characteristics such as low temperature conductivity and corrosion potential, low bacterial contamination, and high biocompatibility [14]. The main complication in the prosthetics was the risk of porcelain fracture or chipping. The short-term clinical results show that this type of constructions may be a viable prosthetic option for the edentulous patient [15–17]. In this clinical case, priority was given to monolithic zirconia implant-supported fixed dental prosthesis with titanium bases. Despite the fact that the full-zirconium construction without bonded titanium bases has the potential of a more favourable soft tissue response, zirconia chipping, as seen in this prosthesis, can occur at a higher rate [18]. Patient's requirement was full-arch fixed restoration up to the second molars. It is well known that excessive cantilever can negatively influence proper biomechanics in implant-supported prosthesis and can result in overloading which may lead to the fracture of the prosthesis and/or abutment screws [14]. In addition, higher length and more quantity of implant supporting a fullarch prosthesis promoted less stress concentration during the simulated load, which is especially important with the use of zirconium. Decreasing the number of implants in rehabilitation is more harmful than decreasing their length for the stress and strain distribution [19, 20]. That is why our team decided for using eight-implant-supported full-arch fixed prosthesis. Advantages of virtual guided technologies for treatment planning are beneficial for the surgeon, orthopaedist-prosthetist, and patient, because it allows performing the operation quickly with maximum accuracy of implant placement into the right position and reducing the trauma of the surgery. Nowadays, CAD/CAM technologies are so far to provide maximum accuracy specifically in edentulous patients. However, the new improvements and trends reduce this gap each time [21, 22]. Implant placement on edentulous jaws is difficult in case of the attaching a surgical template on soft tissues cannot give us accuracy of positioning, even when using intraosseous fixing pins. In this regard, the approach of using strategic teeth in combination with two surgical templates (positioning and full-guided) allows surgery according to preoperative planning. In this case, clinical and CBCT results confirm the fact that the usage of virtual guided technologies can reduce the incidence of complications associated with dental implantation, help the doctor with choosing the most appropriate option of surgery (e.g., flapless method), and reduce the time of operation and healing time after due to minimal trauma of the soft tissues. Dental implant positioning accuracy is an important factor in full-arch implant-supported rehabilitation. However, the choice of retaining type of prosthetic construction is highly important. Cement-retained prosthesis has some advantages like a simpler construction technology, a compensation of placed implants absence of parallel axes, and a passive fitting of prosthesis due to cement space between construction and abutment [5, 12]. However, this type of fixation may provide other complications like the absence of maintenance service possibility and the risk of peri-implantitis development because of excess cement in the peri-implant area, more specifically in soft tissues [23]. Therefore, most clinicians agree that in full-arch implant-supported prosthetics, preference should be given to screw retaining. This type of retaining ensures the maintainability of prosthesis and the possibility of its removal for monitoring and hygienic procedures, and more importantly, screw retaining completely eliminates cement in the peri-implant tissues. The last is most important, because excess cement control is difficult due to fake gingiva on prosthetic construction [24, 25]. On the other side, screw-retained prosthesis may not give passive fitting of construction in case of differences of screwing axes, exactly of them significant divergence. Moreover, at this type of fixation, complications may vary from a fracture of various components in system implant suprastructure to an implant fracture or failure of its osteointegration [6, 10]. In this clinical case, implant placements were made by using surgical template, but this construction may not guarantee implants' parallel axes in need for preparing a screw-retained prosthesis from bone level platform, because of 1–2-degree divergence may result in tension in implant suprastructure system. In this case, four implants were placed in the front side of the maxilla with significant vestibular axes tilt due to bone anatomy specificity. Furthermore, this. situation complicates performing a screw-retained full-arch prosthetic construction from the bone level. The usage of 30-degree multiunit abutments in combination with direct multiunit abutments guaranteed absolutely passive fitting of full-arch prosthesis. The use of multiunit abutments has other advantages. It is well-known that any factor disrupting soft tissue integrity of the biological width may affect the bone level around the implant. Standard protocol of implant-supported rehabilitation includes frequent unscrewing the healing abutment or temporary abutment until the final construction will be retained. Multiple suprastructure unscrewing leads to permanent failure of poor hemidesmosomal soft tissue connection around an implant with following reduction of connective tissue circle. This contributes to a formation of a newer connection and less strong and narrower hemidesmosomal attachment. All of this could be a reason for bone resorption, especially at patients with thin mucosa biotype. Some studies show that multiple abutment screwing-unscrewing series could affect the oral mucosa barrier and could lead to bone loss [26–28]. The meta-analysis by Koutouzis T et al. [29] showed that multiple screwing-unscrewing truly leads to marginal bone loss despite the controversy of results from other studies. In this clinical case, CBCT scanning 1 year after prosthetics shows the consistent bone level around dental implants without remodelling of the peri-implant bone. Moreover, implants were used with six-sided connection and without platform switching system. Intraoperative installation of multiunit abutments allows to seal an implant neck and create a new, stronger, and wider hemidesmosomal connection on the multiunit abutment neck level. Further prosthetic manipulations were made by multiunit abutment level, which is higher than peri-implant bone. This made it possible to avoid multiple screwing/unscrewing by implant neck level and to preserve hemidesmosomal connection. All of this enabled the stability of periimplant bone tissues.

## 7. Conclusion

Dental implant positioning accuracy is an important factor in full-arch implant-supported rehabilitation, especially when the final restoration should be prepared without any ceramic gingiva (“nature-like” teeth). Virtual-guided technologies provide optimal implant placement according to the correct prosthetic position and minimise intraoperative trauma and procedure time.

Screw retaining prostheses have some advantages against cement-retained constructions like an absence of cement in peri-implant tissues and a maintenance service possibility (construction monitoring and its unscrewing, professional hygiene procedures, etc.). The multiunit abutments provide absolutely passive fitting of prosthesis even with significant divergence of placed implants axes.

Intraoperative installation of multiunit abutments allows to seal an implant neck and to create a new, stronger, and wider hemidesmosomal connection on the multiunit abutment neck level and makes it possible to avoid multiple screwing/unscrewing, preserving the stability of peri-implant tissues.
